# Identification of novel genes associated with atherosclerosis in Bama miniature pig

**DOI:** 10.1002/ame2.12412

**Published:** 2024-05-08

**Authors:** Dengfeng Ding, Yuqiong Zhao, Yunxiao Jia, Miaomiao Niu, Xuezhuang Li, Xinou Zheng, Hua Chen

**Affiliations:** ^1^ Laboratory Animal Center Chinese PLA General Hospital Beijing China

**Keywords:** atherosclerosis, candidate genes, genome‐wide linkage analysis, major histocompatibility complex, whole genome sequencing

## Abstract

**Background:**

Atherosclerosis is a chronic cardiovascular disease of great concern. However, it is difficult to establish a direct connection between conventional small animal models and clinical practice. The pig's genome, physiology, and anatomy reflect human biology better than other laboratory animals, which is crucial for studying the pathogenesis of atherosclerosis.

**Methods:**

We used whole‐genome sequencing data from nine Bama minipigs to perform a genome‐wide linkage analysis, and further used bioinformatic tools to filter and identify underlying candidate genes. Candidate gene function prediction was performed using the online prediction tool STRING 12.0. Immunohistochemistry and immunofluorescence were used to detect the expression of proteins encoded by candidate genes.

**Results:**

We mapped differential single nucleotide polymorphisms (SNPs) to genes and obtained a total of 102 differential genes, then we used GO and KEGG pathway enrichment analysis to identify four candidate genes, including *SLA‐1*, *SLA‐2*, *SLA‐3*, and *TAP2*. nsSNPs cause changes in the primary and tertiary structures of SLA‐I and TAP2 proteins, the primary structures of these two proteins have undergone amino acid changes, and the tertiary structures also show slight changes. In addition, immunohistochemistry and immunofluorescence results showed that the expression changes of TAP2 protein in coronary arteries showed a trend of increasing from the middle layer to the inner layer.

**Conclusions:**

We have identified *SLA‐I* and *TAP2* as potential susceptibility genes of atherosclerosis, highlighting the importance of antigen processing and immune response in atherogenesis.

## Graphical abstract

In our previous research, we found that the clinical manifestations of atherosclerosis in Bama minipigs under the same HCFD dietary induction and feeding environment conditions were different (published separately). Three pigs were susceptible to atherosclerosis, and the remaining six pigs were non‐susceptible to atherosclerosis. Excluding other controllable factors, we speculate that genetic factors may play a key role. Liver samples from nine pigs were used for whole‐genome sequencing. According to the different susceptibility of pigs to atherosclerosis, we set some filtering conditions and used whole‐genome linkage analysis to screen for mutations in exon regions that are not easily found. This identification and verification of new atherosclerosis susceptibility genes provides novel ideas for atherosclerosis research and treatment..

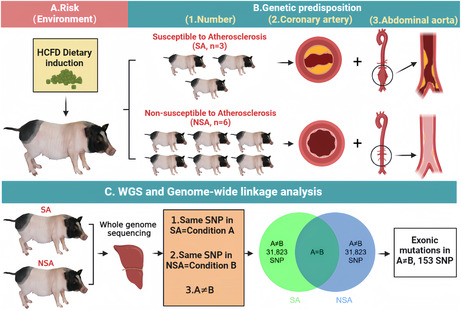

## 1 INTRODUCTION

Atherosclerosis (AS) is a chronic whole‐body disease characterized by lipid infiltration and macrophage‐derived foam cell formation and is the most common cause of cardiovascular disease (CVD).^1^ Coronary artery disease (CAD) is the primary contributor to global mortality, and atherosclerosis emerges as the principal underlying ailment associated with CAD, ischemic stroke, and peripheral vascular disease.^2^ CAD is a troublesome disease with genetic susceptibility, and a person's risk of developing CAD is regulated by the interaction of genetic factors and lifestyle. With the completion of the human genome sequencing project, genome‐wide association studies (GWAS) have become the focus of genetic studies on complex diseases. As of 2021, 321 SNP sites have been found to be significantly related to CAD, and the sample size and number of related sites are still increasing.^3^ Through the analysis of these CAD‐related genetic variants, it was found that more than 90% of the mutation sites are in non‐coding regions of the genome, and all variant sites are common variants and have minor effects.^4^ Although GWAS studies completed so far include a huge number of patients and healthy controls, they clearly cannot reveal all the genetics of CAD. It is reported that genetic factors account for 40% of the pathogenesis of CAD. It then follows that the combined 153 GWAS significant SNP sites can only explain 10.6% of them.^5^ At the same time, with the recent increased use of whole‐genome sequencing (WGS), linkage analysis is again emerging as an important and powerful analysis method for the identification of genes involved in disease etiology, often in conjunction with WGS filtering approaches.^6^ It is currently believed that most of the functional variants are hidden in exons and are caused by low frequency and rare mutations.^7^ Therefore, it is necessary to apply more comprehensive sequencing technologies, especially whole‐exome/whole‐genome sequencing, to discover new and more effective CAD‐related genetic variants.^8^ It has been reported that studying rare coding variants with a large impact on disease can lead to rapid translation from new connections to new methods of treatment.^9^, ^10^ Therefore, combining exon mutations in coding regions with genome‐wide linkage analysis may lead to different new discoveries.

Rat, rabbit, pig, dog and non‐human primate are commonly used model animals in AS research. Hamsters, mice, cat and guinea pig are also used.^11^ Animal models are used in atherosclerosis as an essential tool to improve the understanding of atherosclerotic plaque formation and progression, and animal models of atherosclerosis have the potential to solve the problems of inherent restrictions in human research.^12^ With the emergence of gene knockout mice models, mouse models have gradually become the most commonly used models for AS research.^12^, ^13^ The application of genetically modified mouse models has facilitated preliminary drug efficacy research while at the same time greatly promoting the discovery and interpretation of pathways involved in the occurrence and development of AS.^14^ On the other hand, extrapolation of studies to human disease or as preclinical models to demonstrate the effectiveness of new drugs has been less satisfactory. It has even been questioned whether the mouse model is the same disease as human AS.^14^

The physiological vascular structure of pigs and the distribution, hemodynamics, blood lipid levels, response to a high‐cholesterol diet, AS predilection sites and lesion plaque morphology and structure in pigs are all very similar to humans.^15^, ^16^ The metabolic mechanism of pig lipoprotein, which can cause spontaneous AS and accelerate the formation of AS lesions after a high‐fat and high‐cholesterol diet, is similar to that of humans.^17^ No matter how the porcine AS model is obtained, the pathological characteristics and cellular composition of the arterial lesions, as well as the development process of the lesions, are consistent with human lesions.^18^, ^19^ Experimental studies of human atherosclerosis require appropriate animal models that simulate human physiology and pathology, and swine meet this requirement.^20^ In a preliminary study by our team, feeding Wuzhishan Miniature pigs and Bama minipigs a high‐cholesterol and high‐fat diet (HCFD) resulted in the appearance of plaque lesions in the abdominal and descending aorta and coronary arteries 8 or 9 months later.^15^, ^21^

While the development of atherosclerosis is influenced significantly by environmental factors like diet and smoking, genetic factors also contribute significantly to the risk of atherosclerotic cardiovascular disease. Advances in molecular genetic techniques have shown that genetic disorders significantly influence susceptibility to atherosclerotic vascular disease. Many candidate genes, genetic polymorphisms, and susceptibility loci associated with atherosclerosis have been discovered.^22^ Here, we used WGS data from 9 high‐quality Bama minipigs to perform a genome‐wide linkage analysis. Our analysis identified significant associations with formation of atherosclerotic plaque that implicated swine leukocyte antigen class I (*SLA‐I*), including *SLA‐1*, *SLA‐2* and *SLA‐3*, and transporter associated with antigen processing 2 (*TAP2*). In addition, these genes are all related to antigen presentation. In our study of atherosclerosis, we report, for the first time, that genes related to antigen presentation are susceptibility genes. This suggests that susceptibility genes can provide a new approach to preventing the occurrence of atherosclerosis.

## 2 METHODS

### 2.1 Bama miniature pigs

Nine Bama miniature pigs (male, 6 months old, body weight 12–15 kg) were used. The atherosclerosis pig model was established by feeding the pigs a high‐cholesterol and high‐fat diet (HCFD) for 9 months. Based on pathological changes, the pigs were divided into two groups: the susceptible‐to‐atherosclerosis (SA, *n* = 3) and non‐susceptible‐to‐atherosclerosis groups (NSA, *n* = 6).

### 2.2 Antibodies and reagents

Mouse anti‐SLA‐I MAb was purchased from Bio‐Rad. Rabbit anti‐TAP2 PAb was purchased from Affinity. The Rabbit and Mouse Two‐Step Kit and diaminobenzidine (DAB) were purchased from Zhongshan Jinqiao Biotechnology. All antibodies and reagents were used according to the manufacturer's instructions.

### 2.3 Quantitation of atherosclerosis and pathological changes

Atherosclerotic lesion areas in the abdominal aorta of pigs and vascular pathological changes were recorded.

### 2.4 DNA sample preparation and sequencing

Liver tissue samples were collected from Bama miniature pigs, and total DNA was extracted using the column method.^23^ This project was based on the Next Generation Sequencing analysis platform of BGI Genomics Co. Ltd, with 0.5 μg DNA per sample used as input material for the DNA sample preparations. The manufacturer's recommendations were followed to generate sequencing libraries, utilizing the Annoroad Universal EZ DNA Library Prep kit for MGI V1.0 (CAT: AN210105‐S), Annoroad Adapters for MGI, Set‐T (N1‐N12) (CAT: AN210108), and the Annoroad DNA Cyclization kit for MGI V1.0 (CAT: AN210107‐S). Furthermore, index codes were incorporated to assign sequences to the respective samples. Following the purification process, the PCR library composed of double stranded DNA was untwisted and subsequently transformed into a circular structure, creating single stranded circular DNA. To generate DNA nanoballs (DNBs), the innovative rolling circle amplification (RCA) technique was employed. Subsequently, the DNBs were introduced into the chip by means of the automated sample loading system and securely affixed. The loaded chip containing the DNBs was then subjected to sequencing using the DNBSEQ‐T7 instrument, yielding double ended sequencing reads with a length of 150 bp.

### 2.5 Variation detection and annotation

Paired‐end resequencing reads were mapped to the porcine reference genome, Sscrofa11.1 (Sus scrofa‐NCBI‐NLM[nih.gov]) with BWA.^24^ After obtaining the raw sequencing data in FASTQ format, use BWA software to compare these short sequences with the reference genome, we determined the position of the short sequences on the genome, and assembled the short sequences into a complete porcine reference genome Sscrofa11.1. SAMtools^25^ (Version: 0.1.18) software was used to convert mapping results into the BAM format and to filter the unmapped and non‐unique reads. Duplicated reads were filtered with the Picard package^26^ (picard.sourceforge.net, Version: 1.87). Based on comparison to the reference genome sequence, all potential SNP sites in the entire genome were extracted from it using the mutation analysis software GATK.^27^ Individual SNP detection was performed using Haplotype Caller and GVCF modes, respectively, with SNPs then further filtered based on factors such as quality value, depth, repeatability, etc. to obtain a final high‐resolution Reliability SNP dataset. The VCF files of SNPs were then filtered, and ANNOVAR software^28^ (Version: 2013‐08‐23) and existing genome annotation files were used to annotate the SNPs detected in each sample accordingly. Based on the annotation of the genome, the SNPs were classified into different regions. This classification included exons (which overlap with a coding exon), splicing sites (located within 2 bp of a splicing junction), 5′UTRs and 3′UTRs, introns (which overlap with an intron), regions upstream and downstream (within a region of 1 kb upstream or downstream from the transcription start site), and intergenic regions.

### 2.6 Genome‐wide linkage analysis

Linkage analysis can also provide statistical evidence of the involvement of a variant or gene in disease etiology and can be performed either directly using WGS data or after filtering using data on variants that have been followed up by sequencing across entire families.^6^ Therefore, we used genome‐wide linkage analysis and WGS to identify the potential genes in the atherosclerosis samples. Here, we named the same SNPs in 3 samples in the SA group set A, and the same SNPs in 6 samples in the NSA group set B, and then named the different SNPs between the two groups A and B set C. SNP annotation is described in Supplementary Table S2. In set C, we selected SNPs with mutation types in the exon region, including the 5′ untranslated region (UTR) premature start codon gain variant, missense variant, splice region variant, missense variant/splice region variant, splice region variant/intron variant and splice region variant/synonymous variant. In the SNP annotation file, we mapped the SNPs filtered by the above method to the differential genes.

### 2.7 GO and KEGG pathway enrichment analysis

Gene ontology (GO) and Kyoto Encyclopedia of Genes and Genomes (KEGG) pathway analyses were performed for candidate genes using the R package clusterProfiler.^29^ GO analysis was performed using the EnrichGO function in the R package ‘clusterProfiler’. KEGG analysis was conducted using the EnrichKEGG function of the R package ‘clusterProfiler’. *p* < 0.05 was considered statistically significant. The results of the enrichment analysis were visualized using the R package ggplot2.

### 2.8 Protein structure prediction and PPI analysis

To predict the 3D structure of four candidate genes, we used the open‐source code ofAIphaFold v2.0.^30^ The input was the amino acid sequences of *SLA‐1*, *SLA‐2*, *SLA‐3*, and *TAP2*. The output generated from the model consisted of five PDB format text files. These files contained the predicted structure of the model, referred to as the ‘unrelaxed models.’ However, to refine these predicted structures, an Amber relaxation procedure was employed. This procedure resulted in ‘relaxed models’, which exhibited a more accurate and ordered predicted structure. The relaxed models were further ranked based on their model confidence, measured by the Local Distance Difference test (lDDT) score. These ranked models provided a clearer understanding of the reliability and accuracy of the predictions. To visualize the positions of 8 non‐synonymous SNP substitutions, PyMOL (v.2.3.0) was utilized. These positions were represented as spheres, allowing for a visual representation of the substituted residues. However, it is worth noting that some substituted residues did not appear on the visual surface of the 3D structure model. These residues were categorized as internal residues, implying their location within the structure rather than on its surface. Protein–protein interaction (PPI) analysis was conducted using STRING 12.0 database, (http://string‐db.org/) to identify the protein function and interaction.

### 2.9 Immunohistochemistry

Vascular tissue samples were fixed in 10% formalin, dehydrated, and embedded in paraffin for tissue sectioning.^31^ Immunohistochemistry (IHC) detection of SLA‐I and TAP2 proteins were performed using mouse anti‐SLA‐I MAb (Bio‐Rad 1:50) and rabbit anti‐TAP2 PAb (Affinity 1:100) overnight at 4 °C. After washing three times with PBS, the slides were incubated with secondary antibody or fluorescent secondary antibody at room temperature for 1 h. The slides were subsequently visualized by incubating with DAB for 45 s and counterstained with hematoxylin. After dehydration, clearing, and mounting of the slides, the positive immunoreactivity of the slides was determined by slide scanning system SOS‐40P (Shengqiang Technology, China).

### 2.10 Immunofluorescence

Immunofluorescence (IF) detection of SLA‐I and TAP2 proteins was performed using mouse anti‐SLA‐I MAb (Bio‐Rad 1:1000) and rabbit anti‐TAP2 PAb (Affinity 1:1000) overnight at 4 °C. After washing three times with PBS, the slides were incubated with fluorescent secondary antibody at room temperature for 1 h. After washing away the secondary antibodies with PBS, the slides were mounted with anti‐fluorescence attenuation mounting medium containing DAPI. Fluorescence was observed under an Olympus BX53 fluorescence microscope (Olympus, Japan).

### 2.11 Statistical analysis

Data were presented as means±SD and analyzed by Graphad Prism 9.0. Student's *t* test was used to analyze the difference between two groups. Differences were statistically significant (*) when *p* < 0.05.

## 3 RESULTS

### 3.1 Genetic susceptibility of atherosclerotic lesions

**FIGURE 1:**
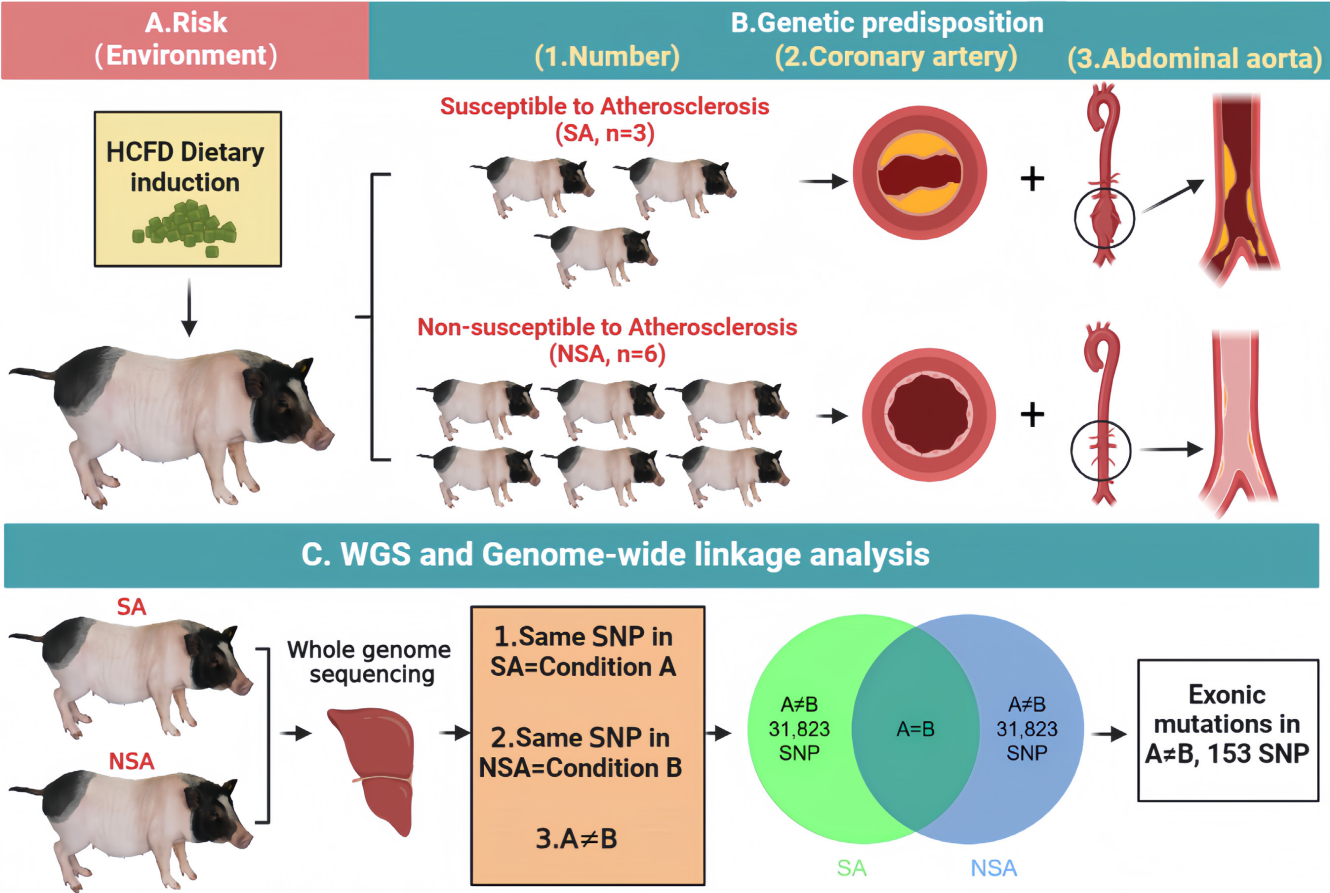
Genetic susceptibility to diet‐induced atherosclerosis and genome‐wide linkage analysis. (A), Environmental factor (high‐cholesterol and high‐fat diet) affects the physiological state of pig blood vessels. (B), Atherosclerotic plaque lesions are more severe in the coronary arteries and abdominal aorta of SA pigs. (C), Sequencing of 9 samples was performed using NGS next generation sequencing, filters based on linkage analysis; 153 susceptible SNP sites were finally obtained.

After nine Bama miniature pigs were fed HCFD under the same environmental conditions for 9 months, intimal thickening was presented at the intima of the anterior descending branch of the left coronary artery and abdominal aorta in the SA group, which appeared to have plaque lesions. Only fatty streak lesions and mild smooth muscle hyperplasia occurred, and no obvious fibrous plaques were formed in the NSA group (Figure 1A,B). We speculate that genetic susceptibility is crucially linked to the formation of intravascular plaques in miniature pigs. Sequencing of 9 samples was performed using next generation sequencing (NGS). Reads were mapped against the porcine reference genome Sscrofa11.1(Susscrofa‐NCBI‐NLM(nih.gov)), yielding a total of 945 million mapped reads, with an average depth of 55.6× covering an average of 96.9% of genome‐wide targets for each accession (Supplemental Table S1). Analysis of this dataset with the Genome Analysis Toolkit (GATK) identified a total of 31 823 target differential SNP loci in SA and NSA groups (Figure 1C; Supplemental Table S2). Single nucleotide polymorphisms (SNPs) are the most abundant form of genetic variation, and thus an ideal genetic susceptibility marker.^32^ We therefore designated the SNPs that differed between the two groups as genetic traits factors (innate susceptibility) for atherosclerosis susceptibility.

### 3.2 Susceptibility genes for *SLA‐1*, *SLA‐2*, *SLA‐3*, and *TAP2*

Given its widespread usage in the investigation of molecular mechanisms and molecular diagnosis of human diseases, whole‐genome exon sequencing has proven to be an efficient method for identifying genetic susceptibility genes and mutation sites associated with complex diseases.^7^ Therefore, we screened SNPs located in exon region variants as susceptibility sites for studying atherosclerotic plaque formation. The number of exonic‐SNPs was 153 (Figure 1; Supplemental Table S3), accounting for 0.48% of differential SNPs, and 102 differential genes were obtained (Supplemental Table S4).

**FIGURE 2:**
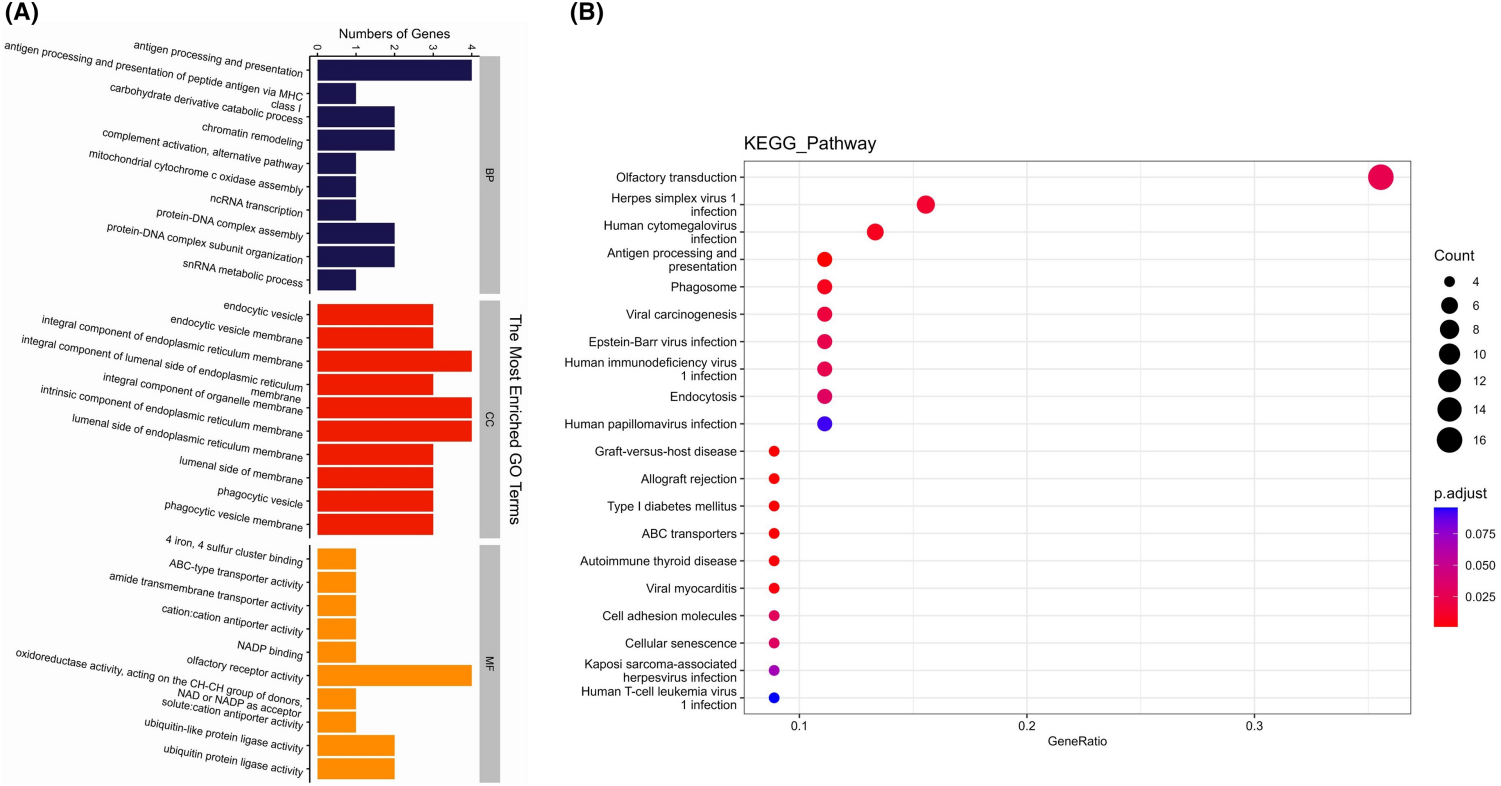
Histogram of GO enrichment analysis and bubble chart of the KEGG pathway enrichment analysis. (A), Categories are divided according to color from top to bottom: Navy blue represents biological process; Red represents cellular component and Orange represents molecular function. (B), The *x*‐axis represents the proportion of genes enriched in the pathway out of the total enriched genes, while the *y*‐axis represents the name of the enriched KEGG pathway. The size of the dots corresponds to the number of genes enriched in the pathway, and the color indicates the *p* value.

In the study of atherosclerosis, a complex disease that is likely caused by mutations in multiple genes, it is important to understand how these genes interact and coordinate with each other. Specifically, we aimed to identify the metabolic pathways and signaling pathways that are most essential in the context of mutant genes. To achieve this, we conducted a significant enrichment analysis using the GO enrichment analysis approach. This analysis focused on three categories: biological process (BP), cellular component (CC), and molecular function (MF). Based on the *p* values obtained from the analysis, we have compiled a list of the top entries, shedding light on the key pathways and functions associated with the shared mutant genes (Figure 2A). The main BP entries that were enriched were antigen processing and presentation. The main CC entries that were enriched included integral components of endoplasmic reticulum membrane and integral components of organelle membrane. Finally, the main MF entries that were enriched related to olfactory receptor activity. Using GO enrichment analysis based on predicted candidate genes, we found a high degree of consistency in the genes mapped in the main BP and CC entries, namely *SLA‐1*, *SLA‐2*, *SLA‐3*, and *TAP2* (Supplemental Table S5). In the SNP annotation file, we found that those genes contain one or more nonsynonymous SNP (nsSNP) leading to amino acid substitutions, those nsSNPs are all located on chromosome 7 and are missense mutation (Table 1). Therefore, we speculate that these genes may be the main susceptibility genes responsible for the inconsistent atherosclerotic plaque formation between SA and NSA groups. In addition, the KEGG database was used to perform pathway enrichment analysis of 102 differential genes. The results showed that those differential genes were significantly enriched in 14 pathways (*p* < 0.05). Here, we list the top 10 pathways through the scatter plot image (Figure 2B). Among them, olfactory transduction, viral infection and antigen processing and presentation are closely related to atherosclerosis susceptibility (Supplemental Table S6).

**TABLE 1:** List of deleterious nsSNPs and amino acid changes in SLA‐1, SLA‐2, SLA‐3, and TAP2.

Chr	Position	Gene	Ret	Alt	NSA group						SA group			Variant feature	Amino acid change
					NSA1	NSA2	NSA3	NSA4	NSA5	NSA6	SA1	SA2	SA3		
7	22824499	SLA‐1	C	**G**	0/0	0/0	0/0	0/0	0/0	0/0	0/1	0/1	0/1	Missense variant	**Ala > Gly**
7	22824966	SLA‐1	T	**A**	0/1	0/1	0/1	0/1	0/1	0/1	0/0	0/0	0/0	Missense variant	**Met > Lys**
7	22824998	SLA‐1	G	**A**	0/0	0/0	0/0	0/0	0/0	0/0	0/1	0/1	0/1	Missense variant	**Gly > Arg**
7	22916775	SLA‐2	C	**T**	0/0	0/0	0/0	0/0	0/0	0/0	0/1	0/1	0/1	Missense variant	**Pro > Leu**
7	22958209	SLA‐2	C	**G**	0/1	0/1	0/1	0/1	0/1	0/1	0/0	0/0	0/0	Missense variant	**Ile > Met**
7	22939351	SLA‐3	A	**T**	0/0	0/0	0/0	0/0	0/0	0/0	0/1	0/1	0/1	Missense variant	**Thr> Ser**
7	25056205	TAP2	G	**C**	1/1	1/1	1/1	1/1	1/1	1/1	0/1	0/1	0/1	Missense variant	**Leu > Val**
7	25056210	TAP2	T	**G**	1/1	1/1	1/1	1/1	1/1	1/1	0/1	0/1	0/1	Missense variant	**Glu > Ala**

*Note*: Comparison of 4 candidate genes in SA and NSA groups. Base and amino acid change are denoted in bold. 0/0: homozygous allele same as the reference; 0/1: heterozygous variant; 1/1: homozygous variant.

### 3.3 Candidate gene function prediction

**FIGURE 3:**
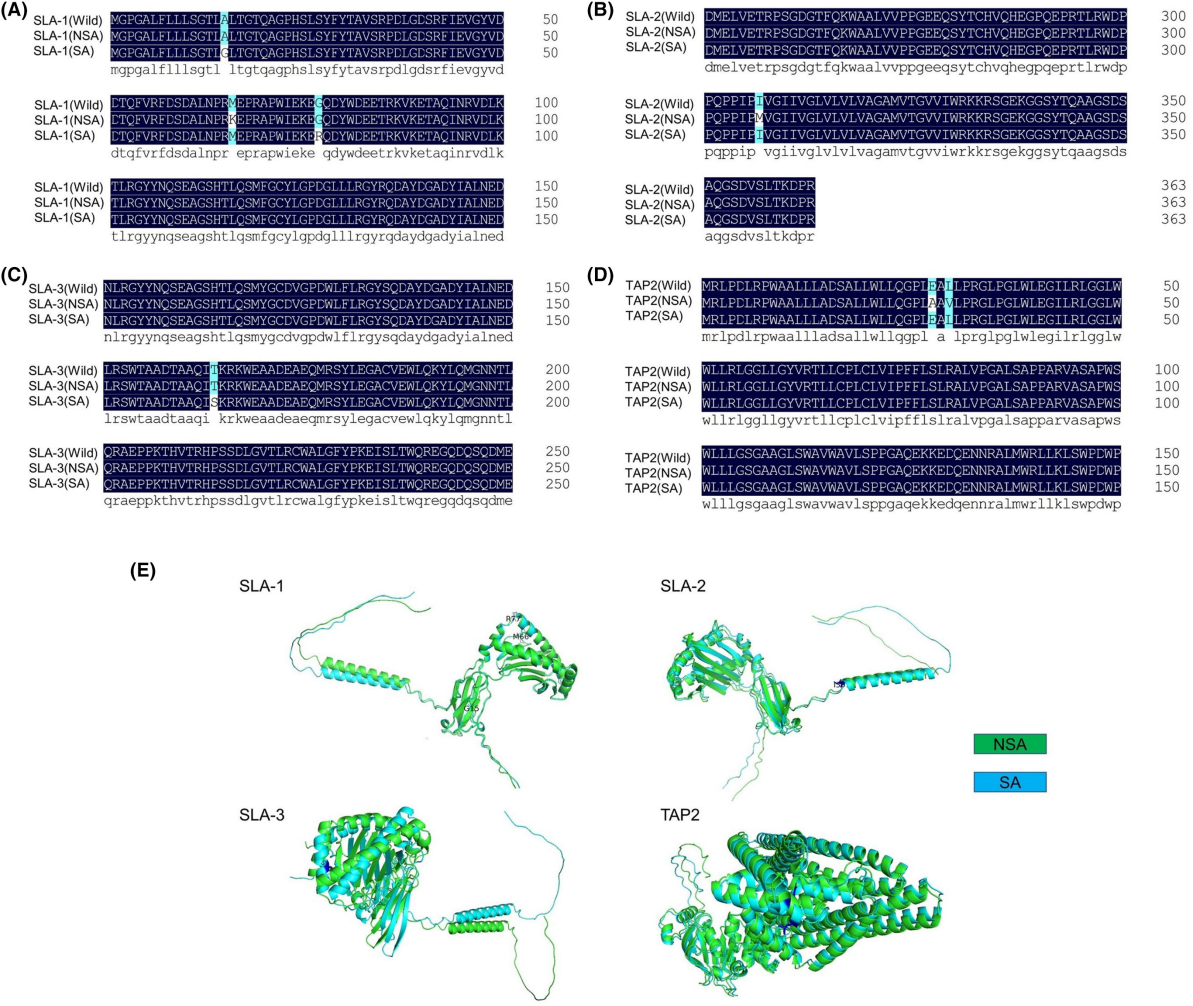
Candidate gene function prediction. (A), Changes in amino acids in the primary structure of SLA‐1 protein. (B), Changes in amino acids in the primary structure of SLA‐2 protein. (C), Changes in amino acids in the primary structure of SLA‐3 protein. (D), Changes in amino acids in the primary structure of TAP2 protein. (E), Comparison of the tertiary structure of SLA‐1, SLA‐2, SLA‐3, and TAP2 protein in NSA and SA groups.

Protein structure and gene structure are closely related to gene function.^33^ To further understand the consequences of nsSNPs, we performed an in‐depth analysis of their primary protein structure. We obtained the gene sequence of the candidate gene from the NCBI database, replaced the nsSNP site bases according to Table 1, and then obtained the protein sequence using DNAMAN. Using our alignment, changes in protein primary structure and amino acid sequence were detected (Figure 3A–D; Supplemental Figure S1). The dark blue background represents the normal amino acid sequence, and the light blue background represents the amino acid changes caused by SNP mutations. Therefore, we further used the open‐source code of AIphaFold v2.0.to predict the impact of one or multiple amino substitutions on the tertiary structure of SLA‐1, SLA‐2, SLA‐3 and TAP2 protein products. The three‐dimensional protein structure predicted by Alphafold found that the three mutations of SLA‐1 all occurred in the non‐return coil, and there was no obvious change in the protein structure of SLA‐1 (NSA) and SLA‐1 (SA). The SLA‐2 mutation occurred in the helix on the top and there was no obvious change in the protein structure of SLA‐2 (NSA) and SLA‐2 (SA). The SLA‐3 mutation occurred on the helix, and the protein structure of SLA‐3 (NSA) and SLA‐3 (SA) changed slightly. For TAP2, both mutations occurred on the same helix, and there was no obvious change in the protein structure of TAP2 (NSA) and TAP2 (SA) (Figure 3E).

### 3.4 TAP2 proteins were detected in the coronary artery and PPI analysis

**FIGURE 4:**
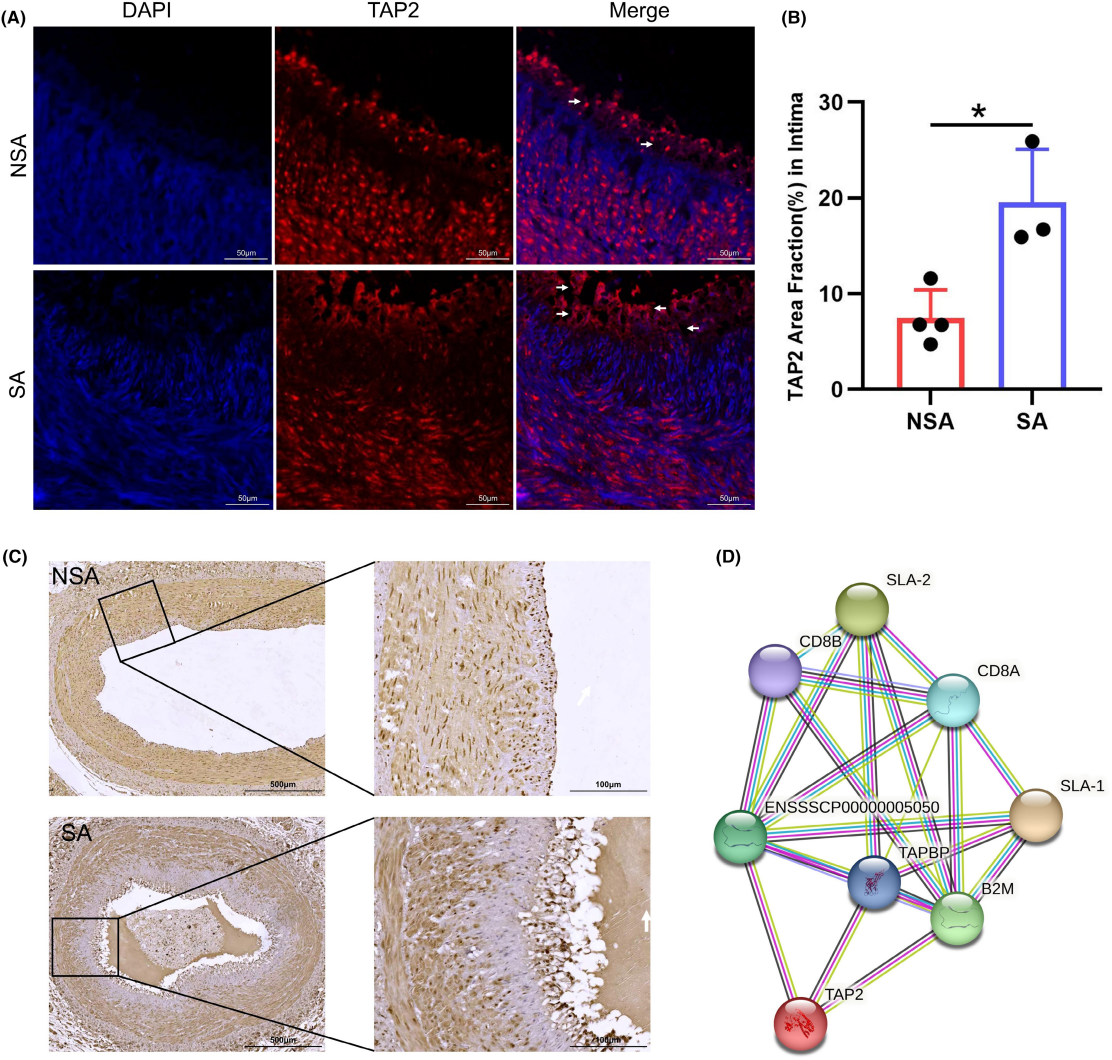
Expression of TAP2 protein in the coronary artery and PPI analysis. (A), TAP2 immunofluorescence in coronary artery of NSA and SA groups. Blue fluorescence represents the nucleus, and red fluorescence represents TAP2. (Scale bars: 50 μm). (B), The fluorescence area values of the two groups were statistically different (*p < 0.05). (C), TAP2 immunohistochemistry in coronary artery of NSA and SA groups. (Scale bars: 500 μm, 100 μm). (D), Protein–protein interaction network of candidate genes. The red line indicates evidence of gene fusion; the black line indicates co‐expression evidence; the blue line indicates co‐occurrence evidence; the light blue line indicates curated database evidence; the purple line indicates experimental evidence; the yellow line indicates text mining evidence; the green line indicates gene neighborhood evidence.

Swine MHC class I is commonly known as swine lymphocyte antigen (SLA‐I), and it plays a crucial role in the swine genome. The swine genome consists of three classical *SLA‐I* loci, namely *SLA‐1*, *SLA‐2*, and *SLA‐3*, all of which display dominant expression. These loci are significant for understanding the immune response and genetic variations in pigs. The presence of multiple *SLA‐I* loci in the swine genome is indicative of the complexity and diversity of the swine immune system. By studying these loci and their expression, researchers can gain insights into the adaptive immune responses and genetic diversity within the swine population, and knowledge about *SLA‐I* loci can assist in enhancing the understanding of swine immune responses, disease resistance, and the development of vaccines.^34^ Due to the high homology of these three genes, there is currently only one SLA‐I antibody produced by Bio‐Rad. To determine whether SLA‐I and TAP2 proteins can be detected in the coronary artery, we performed IHC and IF experiments. The results showed that compared with the NSA pigs, the expression of TAP2 protein in the coronary intima layer of the SA pigs was significantly increased (white arrow), and obvious cell deformation was also detected (Figure 4A). By counting the fluorescence areas in the two groups, it was found that there was a statistical difference between them (*p* < 0.05; Figure 4B). Slight thickening of the endothelial cell layer was found in the blood vessels of the NSA pigs. Plaque‐like tissue appeared in the center of the blood vessels of the SA pigs. The endothelial cell layer was thickened and varied significantly. *TAP2*, a transporter associated with antigen processing, was detected in the coronary artery. Comparative analysis with NSA pigs found that the expression of TAP2 protein in the thickened endothelium of blood vessels in SA pigs increased, and there was a tendency for the outer layer to shift to the inner layer. TAP2 protein was involved in the development of atherosclerosis, as expected (Figure 4C). However, we did not detect the presence of SLA‐I protein in coronary artery using the method of Xiang Liu et al^35^ (Supplemental Figure S2).

To further predict the correlation between SLA‐I and TAP2 proteins, a PPI network of those genes was constructed using STRING. The results of the PPI suggest that there may be a potential association between these proteins. If so, TAP2 is required to bind to other related proteins involved in antigen presentation and transport [such as SLA‐I, CD8^+^ T cell, TAP‐associated glycoprotein (TAPBP) and Beta2‐microglobulin (B2M)] to function. (Figure 4D).

## 4 DISCUSSION

Atherosclerosis (AS) is a chronic whole‐body cardiovascular disease that is influenced by various factors, including genetics and the environment. It typically takes several decades for clinical complications to arise in humans. Several well‐known risk factors contribute to the development of AS, including hypercholesterolemia, hypertension, diabetes, smoking, and chronic inflammation of the blood vessels.^36^ The latest research shows that viral infection is also a causative factor in AS. Viruses can initiate atherosclerosis through two pathways. They can directly infect vascular cells, leading to inflammation in the endothelium and smooth muscle cells. Alternatively, they can indirectly affect atherogenesis by infecting non‐vascular cells and inducing systemic inflammation.^37^ We chose Bama miniature pigs, which are closer to human physiology and lipid metabolism, as an animal model, which is an important prerequisite for better research on AS. In this study, the conditions of the Bama minipigs themselves (breed, source, gender, age, microbial quality) and the experimental induction factors (rearing environment, induction feed, feeding and management) were consistent. However, the early HE pathology results showed that there was a difference between SA and NSA groups. The degree of plaque lesions was different. As we all know, pathological methods are more accurate and reliable than imaging diagnostic methods to evaluate the severity of AS lesions in animals. This suggested that the differences in lesions were mainly due to differences in genetic characteristics. It is currently believed that most of the functional variants are hidden in exons and are caused by low frequency and rare mutations.^7^ We performed linkage analysis using exon mutations from 9 high‐quality Bama minipigs and identified 102 candidate genes associated with atherosclerotic plaque formation. We then used GO and KEGG pathway enrichment analysis to reduce the number of candidate genes down to 4 genes. Moreover, differences in protein structure of candidate genes between SA and NSA groups were found, and TAP2 protein was detected in the coronary artery. Mechanistically, under the same dietary induction conditions, differences in the SNP sites of *SLA‐I* and *TAP2* genes between SA and NSA groups seem to be an important factor in the formation of atherosclerotic plaques; this needs to be further clarified in future studies (Figure 1).

The rising accessibility of omics data, including genomes, transcriptomes, proteomes, and metabolomes, has created an ongoing need for techniques capable of extracting valuable biological insights from these intricate datasets. Gene Set Enrichment Analysis has emerged as a widely used and effective tool for analyzing genome data.^38^ Through gene enrichment analysis, we found that gene functions and pathways are mainly concentrated in antigen presentation, and we further reduced the number of candidate genes to four (Figure 2; Table 1). In atherosclerotic mice, single‐cell sequencing found that genes controlling antigen presentation were involved in the control of atherosclerosis‐associated inflammation.^39^ We speculate that these four antigen‐presenting genes, which have received little attention to date, may be closely related to the development of AS. Genome‐wide association studies have identified an association between a nonsynonymous SNP and various human diseases.^40^, ^41^ We focused on candidate genes that carry nonsynonymous coding SNPs that almost certainly affect protein function (Table 1, Figure 3). Changes in the helical conformation of porcine *SLA‐I* and *TAP2* gene are related to viral infection and inflammation.^42^, ^43^ Thus, based on the close relationship between *SLA‐1* and *TAP2* genes and immune response and inflammation, we believe that enhanced understanding of these genes may be a promising target for clinical prediction of AS. Swine MHC class I, also known as swine lymphocyte antigen (*SLA‐I*), is composed of three main loci (*SLA‐1*, *SLA‐2*, and *SLA‐3*), which are highly prevalent in the swine genome and show dominant expression.^34^ Recent studies found that the most important gene for immune responses to swine infectious diseases and vaccines remains *SLA‐I*.^44^ Indeed, MHC has been shown to be a major genetic determinant of atherosclerosis, mediating the development of inflammation in atherosclerosis.^45^, ^46^ Our results reveal that *SLA‐I* may play a key role in atherosclerosis as an antigen‐presenting gene (Figures 2 and 3).

The transporter associated with antigen processing (TAP) has an essential function in transporting peptides from the cytosol to the endoplasmic reticulum (ER) lumen in the major histocompatibility complex (MHC) class I antigen presenting pathway. After reaching the ER lumen, the peptides are then loaded onto MHC class I molecules.^47^ TAP2 is a transporter associated with antigen processing, and its genetic polymorphisms are related to various diseases.^48^, ^49^, ^50^ It is worth noting that TAP2 protein was detected by IHC and IF, and showed a trend towards decreased expression in the middle layer and increased expression in the intimal layer in SA swine coronary arteries (Figure 4A–C), suggesting that TAP2 is involved in the formation of atherosclerosis and may mediate the intravascular inflammatory process. Unfortunately, we did not detect the presence of SLA‐I protein. The CD8^+^ T cell antiviral immunity greatly relies on MHC‐I, which plays a crucial role in processing and presenting intracellular antigen peptides.^51^ Recent reports suggest that CD8^+^ T cells are also important modulators of atherosclerosis. CD8^+^ T cell activation and CD8^+^ T cell mediated macrophage death caused progression towards high‐risk plaques.^52^, ^53^ Moreover, the STARNET study indicated that CD8^+^ T cells indirectly communicated with TAP2 through TAPBP and B2M, which suggested that there may be an immune system imbalance in the atherosclerosis process. There are reports confirming that an imbalance between immune inflammation and immune regulation contributes to the development of atherosclerosis, and the activated immune system potentially accelerates atherosclerosis, and atherosclerosis activates the immune system, creating a vicious circle.^54^, ^55^ The candidate genes we obtained happened to be genes related to immune response. This discovery makes the study of atherosclerosis more interesting. Changes in the expression of TAP2 protein in the inner membrane layer are strong evidence. These results are convincing. In early cancer research, M Vitale et al found that mutations in HLA‐class I antigens and TAP2 may indicate evasion of host immune pressure, and the accumulation of defects in antigen processing and presentation may in turn lead to reduced recognition by CD8^+^ T cells.^56^ Further research is needed to elucidate the molecular mechanisms by which *SLA‐I* and *TAP2* participate in the progression of AS.

In summary, we filtered the susceptibility SNP sites associated with atherosclerosis through genome‐wide linkage analysis, and further used bioinformatic tools to filter and identify underlying differential genes. Our finding that the MHC region genes *SLA‐I* and *TAP2* play an important role in immune response highlights the screening role and preventive significance of *SLA‐I* and *TAP2* genotyping in early clinical atherosclerosis. Due to the small number of samples in this study, we are using pigs to further verify this finding, which may also provide new ideas for the prediction and prevention of genetic susceptibility to atherosclerotic diseases.

## AUTHOR CONTRIBUTIONS

Hua Chen and Dengfeng Ding designed and directed the project and analyzed data. Dengfeng Ding, Yuqiong Zhao, Yunxiao Jia, Miaomiao Niu, Xuezhuang Li, Xinou Zheng, and Hua Chen performed the experiments. Chen Hua is the guarantor of this work, and takes responsibility for the integrity of the data and the accuracy of the data analysis.

## FUNDING INFORMATION

This work was supported by the Special Scientific Research Project of Army Laboratory Animals (No. SYDW [2020]01) and National Natural Science Foundation of China, No. 32370568.

## CONFLICT OF INTEREST STATEMENT

The authors declare no competing interests.

## ETHICS STATEMENT

All animal experiments were conducted in compliance with the guidelines set by the Institutional Animal Care and Use Committee of Chinese PLA General Hospital (ID: 2018‐D14‐26).

## Supporting information


Supplemental Figure 1.

Supplemental Table 1.

Supplemental Table 2.

Supplemental Table 3.

Supplemental Table 4.

Supplemental Table 5.

Supplemental Table 6.
