# Supplementary material for: Identification of novel genes associated with atherosclerosis in Bama miniature pig
Source: Animal Model Exp Med. 2024 May 8;7(3):377–87. doi: 10.1002/ame2.12412 (PMC11228093; doi:10.1002/ame2.12412)
Supplement: Supplementary file 1 — Supplemental Figure 1. [file AME2-7-377-s007.pdf]

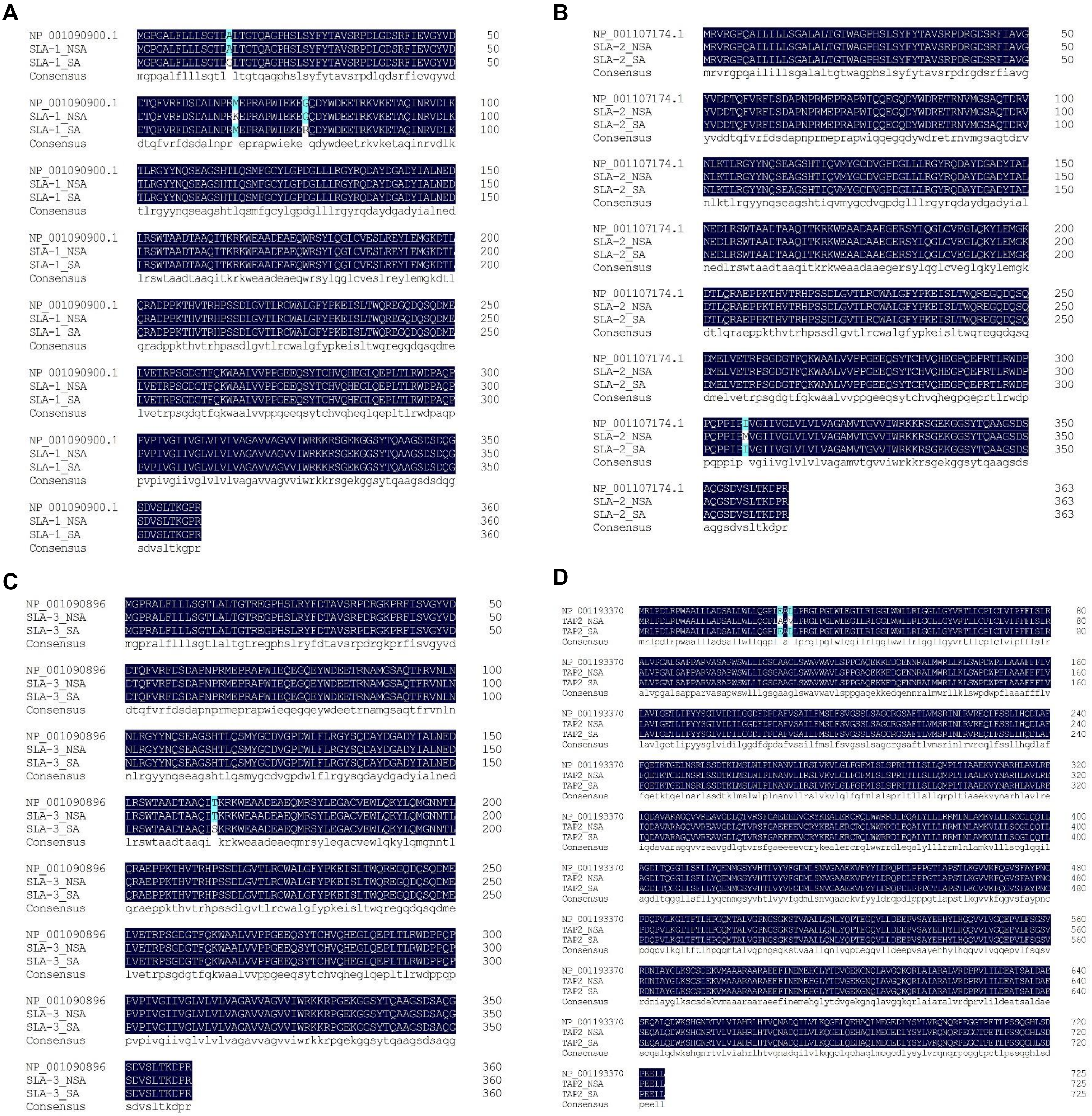

Supplemental Figure 1 Candidate gene function prediction. (A) Changes in amino acids in the primary structure of SLA-1 protein. (B) Changes in amino acids in the primary structure of SLA-2 protein. (C) Changes in amino acids in the primary structure of SLA-3 protein. (D) Changes in amino acids in the primary structure of TAP2 protein.

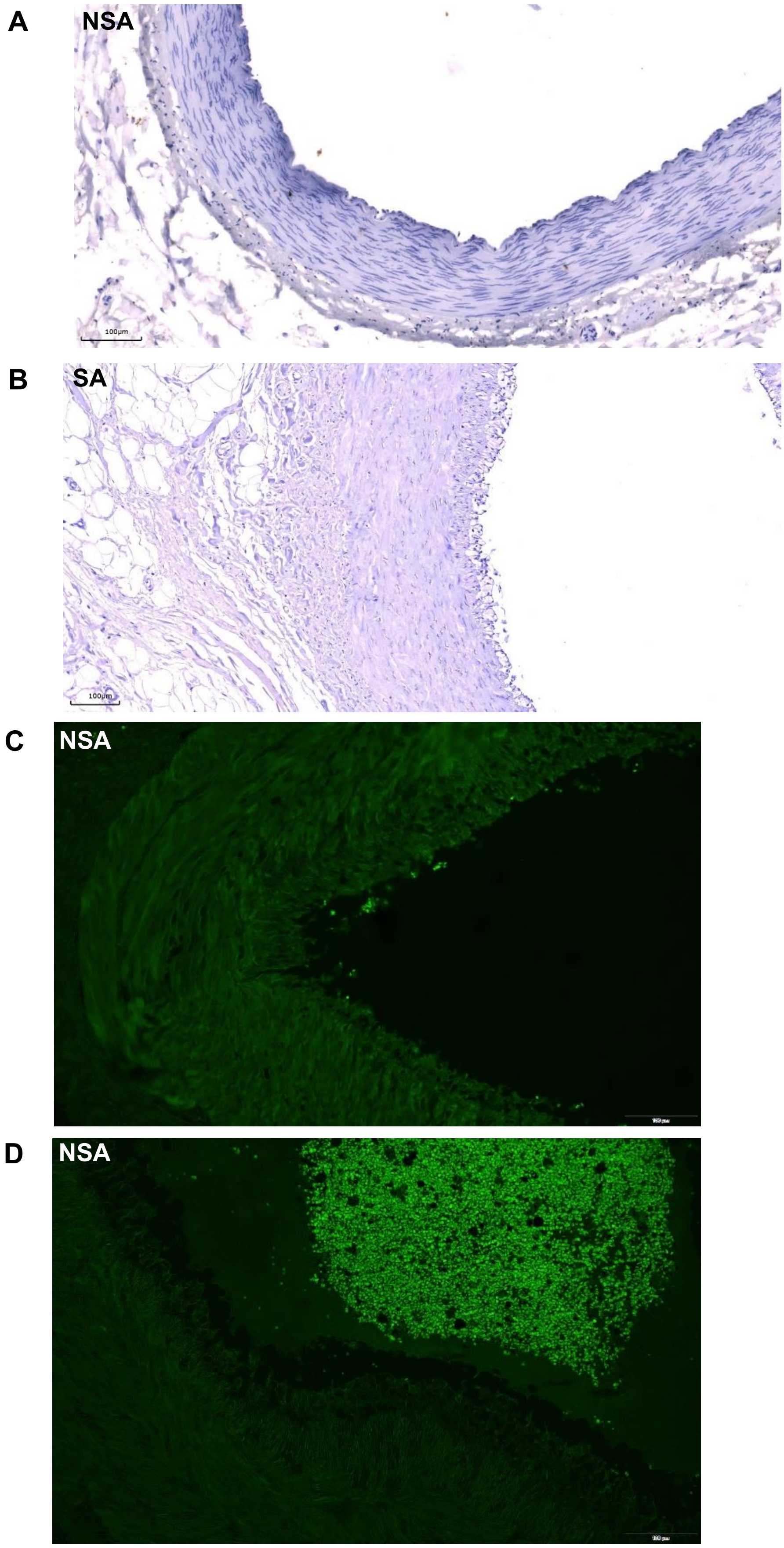
A
NSA
100μm
B
SA
100μm
C
NSA
D
NSA

Supplemental Figure 2 SLA-I immunohistochemistry and immunofluorescence in coronary artery of NSA and SA groups. No expression of SLA-1 protein was detected in coronary artery. Scale bars: A, B, C and D 100μm.
